# Contingent negative variation as an evaluation indicator of neurocognitive disorder after traumatic brain injury

**DOI:** 10.3389/fpsyt.2023.1255608

**Published:** 2023-12-19

**Authors:** Xindi Ling, Shujian Wang, Shengyu Zhang, Wen Li, Qinting Zhang, Weixiong Cai, Haozhe Li

**Affiliations:** ^1^Shanghai Key Lab of Forensic Medicine, Key Lab of Forensic Science, Ministry of Justice, Shanghai Forensic Service Platform, Academy of Forensic Science, Shanghai, China; ^2^Department of Forensic Medicine, School of Basic Medical Sciences, Fudan University, Shanghai, China

**Keywords:** neurocognitive disorder, traumatic brain injury, event-related potential, contingent negative variation, evaluation indicator

## Abstract

**Introduction:**

Neurocognitive disorders are commonly observed in patients suffering from traumatic brain injury (TBI). Methods to assess neurocognitive disorders have thus drawn the general attention of the public, especially electrophysiology parameter such as contingent negative variation (CNV), which has been given more emphasis as a neurophysiological marker in event-related potentials (ERPs) for diagnosing a neurocognitive disorder and assessing its severity. The present study focused on the correlations between CNV parameters and levels of daily living activities and social function to explore the potential of CNV as an objective assessment tool.

**Methods:**

Thirty-one patients with a diagnosis of neurocognitive disorder after a TBI according to ICD-10 were enrolled as the patient group, and 24 matched healthy volunteers were enrolled as the control group. The activity of daily living scale, functional activities questionnaire, social disability screening schedule, and scale of personality change following TBI were used to assess daily living activity and social function.

**Results:**

The scale scores in patients were significantly higher than those in controls. Maximum amplitudes before S2 and during the post-imperative negative variation (PINV) period were also significantly higher in the patient group compared to the control group and were positively correlated with four scale scores. The duration of PINV at Fz and Cz was significantly shorter in the patient group than in the control group. The CNV return to baseline from a positive wave at electrode Fz and Cz occurred significantly earlier in the control group than in the patient group, while at Pz, the result showed the opposite.

**Conclusion:**

Lower amplitudes of CNV were associated with more severe neurocognitive disorder and greater impairments in daily life abilities and social function. The duration of PINV and the latency of returning to baseline from a positive wave were correlated with the neurocognitive disorder to some extent. CNV could be used as an objective, electrophysiology-based parameter for evaluating the severity of the neurocognitive disorder and personality changes after TBI.

## Introduction

Traumatic brain injury (TBI) is a worldwide health concern often caused by traffic accidents (1), which might be a modifiable risk factor for dementia and neurodegeneration (2). In addition to causing substantial brain damage, TBI can lead to mental issues such as cognitive and memory impairment, personality changes, and psychotic symptoms. Studies have shown that up to 49% of patients with severe and moderate TBI, as well as 34% of those with mild TBI (mTBI), experience neurocognitive disorder (3). The global annual number of patients presenting with concussion after an mTBI is approximately 55.9 million (4). Despite the high prevalence of TBI-related neurocognitive disorders, follow-up assessments of TBI patients are often neglected due to economic considerations. As a result, many people suffer from long-term disability and morbidity. Therefore, advancing research on post-TBI neurocognitive disorders to address this urgent and important global issue is essential.

Computer tomography (CT) scanning and magnetic resonance imaging (MRI) are common imaging modalities for diagnosing TBI. CT is primarily used to identify intracranial injuries but cannot exclude TBI (5). Although advanced MRI techniques such as diffusion tensor imaging are sensitive in detecting axonal injury and structural abnormalities in the brain, they have limited utility in assessing functional injury following TBI (6). In contrast, altered and compensational brain activity can be detected through functional MRI (fMRI), revealing the effect of TBI on certain brain functions to some extent (7).

Recently, event-related potentials (ERPs) have gained increasing attention as electrophysiological tests that can quantify and directly reflect neural activity. P300, mismatch negativity (MMN) and contingent negative variation (CNV) are the most widely used neurophysiological markers in EPRs. CNV is a composite slow wave known as the earliest cognitive potential, which reflects the reciprocal effect of a flashlight and short sound stimulus (8). Two negative-going components are found in CNV: the first is evoked by auditory warning (S1) and is related to orienting, while the later component, known as the “readiness potential,” precedes spontaneous movement and is associated with the imperative stimulus (S2) (9). Furthermore, a hypothesis was provided to suggest that the abnormal CNV reflects the function of the frontal lobe in maintaining cognitive processing (10). Thus far, despite some progress in ERPs and cognitive impairment (11, 12), the correlation between TBI and neurocognitive disorder remains relatively unexplored. Most current studies have focused on P300 and MMN after a TBI (13–15), with few studies exploring CNV and remaining debating on the perceptual and cognitive functions of CNV. Previous studies have examined the correlation between CNV and personality change and psychotic traits in patients with depression and schizophrenia, showing that the lower amplitude of CNV is positively correlated with self-transcendence scores, which may be associated with psychosis and personality disorders (16, 17). Reduced amplitude of CNV was observed in patients with schizophrenia and patients with severe level of depression (18, 19). Thus, our study speculated that there may also exist relationship between CNV and social functioning, personality traits, and emotions in patients after a TBI. Besides, unlike P300 and MMN, which focus on a single stimulus, CNV is elicited by both visual and auditory stimuli and might provide a more comprehensive assessment of mental disorders following TBI. Therefore, further investigation into CNV is needed to assess its utility in evaluating symptoms, particularly neurocognitive disorders, after a TBI.

Current developments in TBI and its long-term sequelae have underscored the importance of investigating the correlation between daily life abilities, social functioning, and primary trauma (20, 21). Therefore, our study proposed the hypothesis that variations in the subcomponents of EPRs, in particular CNV will differ between healthy subjects and patients suffering from sequelae of TBI and that degree of this aberrance will relate to the grade of the neurobehavioral disturbance. Based on the hypothesis, this study proposed CNV as an objective technique for assessing the severity of neurocognitive disorder after a TBI.

## Materials and methods

### Participants

Based on the International Statistical Classification of Diseases and Related Health Problems, Tenth Revision (ICD-10), cases with a diagnosis of neurocognitive disorder after a TBI (*n* = 31) were enrolled and compared to matched healthy controls (*n* = 24). The inclusion criteria were as follows: (1) aged 18–60 years, (2) right-handed, (3) met the ICD-10 diagnostic criteria for neurocognitive disorder after a TBI, and (4) recovered for at least six months after TBI and received clinical care. Cases were excluded if the participant (1) had experienced cranial trauma before presenting TBI or had other serious physical illnesses such as epilepsy and encephalitis, (2) had a history of mental disorders or had family members with mental disorders, (3) had a history of chronic alcohol, drug, or other psychoactive substance abuse, or (4) lacked consensus. The selected healthy controls met the exclusion criteria mentioned above and the inclusion criteria as follows: (1) aged 18–60 years, (2) right-handed, and (3) without neurocognitive disorders. All participants voluntarily participated in the study.

This study received approval from the ethics committee of the Academy of Forensic Science. All methods and procedures of this study were conducted in accordance with the Declaration of Helsinki and other relevant national and international standards for human research.

### Scale assessment

Demographic and experimental data were collected by self-designed research forms. Before the CNV examination was performed, the participants were assessed using the activity of daily living (ADL) scale, functional activities questionnaire (FAQ), social disability screening schedule (SDSS), and scale of personality change following TBI. The ADL, FAQ, SDSS scale, and scale of personality change following TBI were filled out by the researchers according to the patients’ clinical records and researchers’ interviews. An ADL score > 16 indicated impairment in daily life abilities, with the severity of the impairment corresponding to the score. A FAQ score > 5 indicated problems with independence in family and society. A total SDSS score > 2 indicated social dysfunction. The scale of personality change following TBI is a 19-item scale used to indicate the personality change of the subject. A total score > 7 indicated problems with personality change (22, 23). The ADL, FAQ, SDSS scale, and scale of personality change following TBI were filled out by the researchers according to the patients’ clinical records and researchers’ interviews. Basic demographic information and clinical history of the participants were also collected.

### Procedures of the CNV paradigm

CNV waves were recorded by a Neuroscan device (Australia, Compumedics). During the study, the subjects were seated in a dark and quiet room with an ambient temperature of 22–24°C. The subjects were instructed to be relaxed, stay awake, and focus on the screen. ERPs were recorded according to the 10–20 system. The reference electrodes were linked to the mastoid process. The impedance of Ag/AgCl surface electrodes was maintained below 5 kΩ. The classical CNV experimental model was utilized in this study, which involved presenting an auditory warning (S1) as a 50 ms pure tone at 1000 Hz and 90 dB, followed by the imperative stimulus (S2) after a 1,500-ms interval. S2 was presented as a flashing light that disappeared automatically after 200 ms. Participants were instructed to focus on the computer screen, maintain concentration, and press a button immediately upon seeing the flash. No response was required for S1. Each period from S1 presentation to the button press was defined as a trial. The interval between each trial was randomly set to 2–5 s to eliminate habituation effects on attention. A total of 30 trials were completed by each participant.

**Figure 1:**
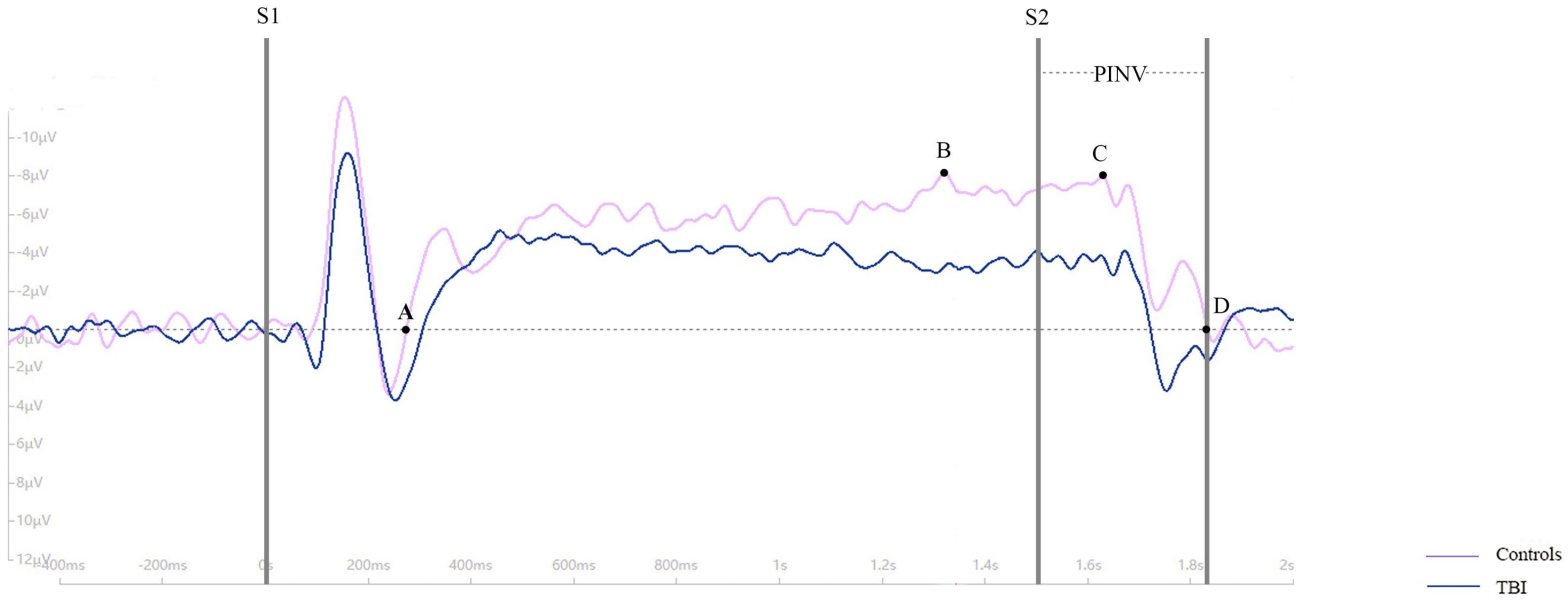
The points A, B, C, D and the duration of PINV in the EEG topography maps.

**Figure 2:**
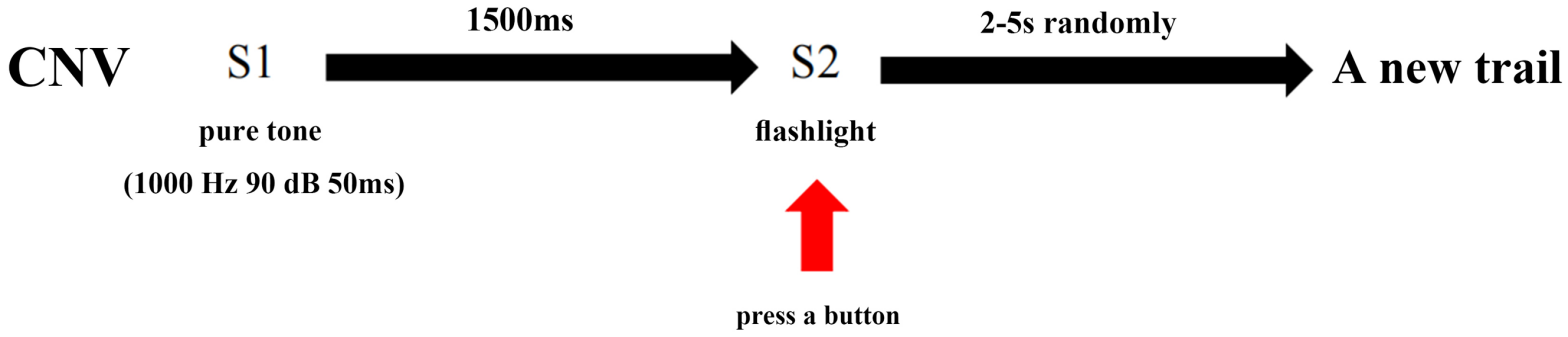
Procedures of the CNV paradigm.

CNV is a negative wave that can be recorded using electroencephalography (EEG). For this study, three scalp sites (Fz, Cz, and Pz) situated over the midline were selected for EEG recording. Bilateral retroauricular mastoid processes were chose as the reference electrode and epicenter of the forehead was chosen as the ground electrode. The baseline period was set as 200 ms before S1, and the period from −200 ms to 2,500 ms was continuously recorded. The study designated point A as the point where the CNV returned to baseline from a positive wave, point B as the maximum amplitude before S2, point C as the maximum amplitude during the period of post-imperative negative variation (PINV), and point D as the point where the CNV returned to baseline from a negative wave. The duration of PINV was the period between S2 and point D. The maximum amplitude of the CNV before S2 and during PINV, as well as the duration of the PINV period, were recorded and analyzed. For a clearer description of the parameters taken in our study, four point and the PINV period has been marked in Figure 1. The procedure of the CNV paradigm is shown as a flowchart in Figure 2.

### Statistical analysis

ERPs data were collected and processed by Curry Neuroimaging Suite (version 7.0). Baseline calibration, parameters filtering, and artifact reduction were conducted before scanning the data. The stimulus artifact of electro-oculogram and electromyography, high-amplitude noise (≥ 75 μV), 50 cycle, and bad block were removed before analysis. The instrumental noise and physiological interference from the body were processed by denoising algorithms. SyAmps2/RT was chosen as the amplifier. The sampling rate was set at 1 kHz. The passband parameter was set at 0.1 Hz-200 Hz.

In this study, data were analyzed using IBM Statistical Product and Service Solutions, Version 22.0 (IBM SPSS 22.0). Recorded data were presented in the form of mean ± standard deviation (SD). All data were corresponded to normal distribution. Chi-square tests were employed to compare categorical data and Student’s *t*-test were employed to compare variable data, respectively, between patients with the neurocognitive disorder after a TBI and matched controls. Pearson’s correlation analysis was used to calculate the correlations between CNV parameters and scale scores. The level of significance was set to *p* < 0.05.

## Results

### Demographic and clinical characteristics

Table 1 indicates that there was no statistically significant difference in gender, age and education level between TBI patient and control groups. The ADL, FAQ, SDSS, and personality change scale scores were significantly higher in the TBI patient group than in the control group (*p* < 0.001).

**Table 1:** Demographic and clinical characteristics between patients with neurocognitive disorders after TBI and controls.

Parameter	TBI (*n* = 31)	Controls (*n* = 24)	Value of *p*
Gender			0.164^a^
Male	14	15	
Female	17	9	
Age in years	36.90 ± 10.81	32.96 ± 12.10	0.251
Education in years	12.81 ± 2.50	14.77 ± 3.66	0.123
ADL scale scores	19.16 ± 4.20	14.58 ± 0.58	<0.001^b^
FAQ scale scores	5.65 ± 2.51	1.38 ± 1.17	<0.001^b^
SDSS scale sores	2.94 ± 2.35	0.17 ± 0.38	<0.001^b^
Personality change scale sores	10.89 ± 6.00	3.04 ± 1.57	<0.001^b^

Except gender, other data were presented as mean ± SD. ADL, activity of daily living; FAQ, functional activities questionnaire; SDSS, social disability screening schedule; TBI, traumatic brain injury. ^a^Chi-square test; other statistics: Student’s *t*-test. ^b^Statistically significant differences.

#### CNV parameters

**Figure 3:**
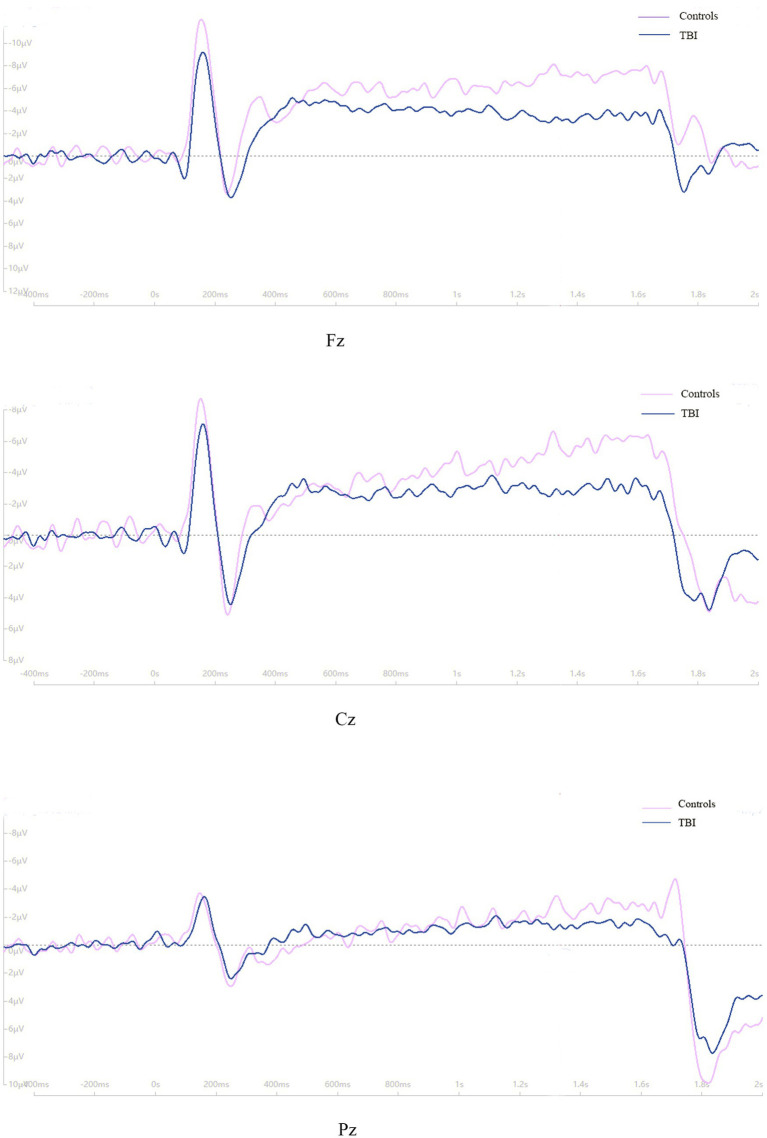
The grand average ERPs response between patients and healthy controls.

**Figure 4:**
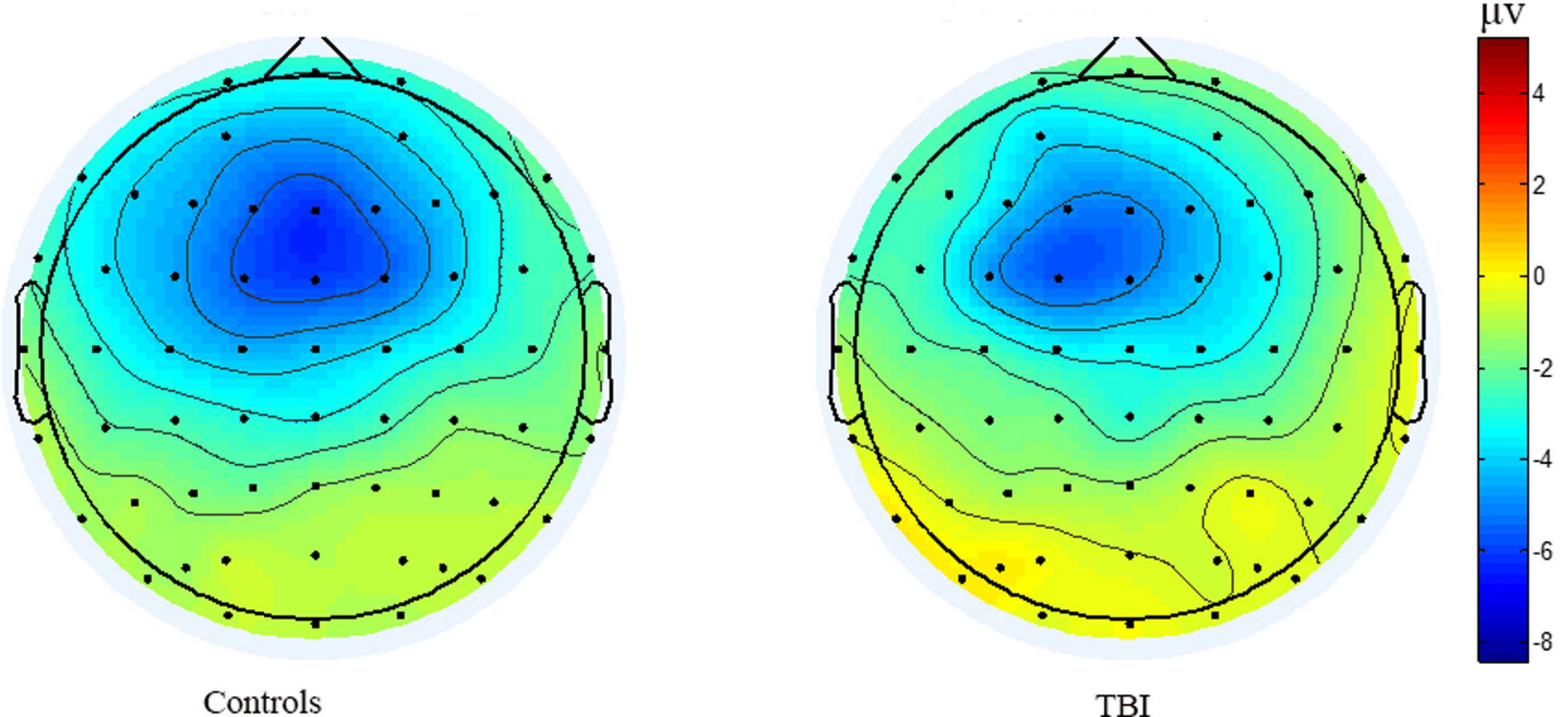
The topographic distribution of the grand average CNV amplitudes between patients healthy controls.

**Figure 5:**
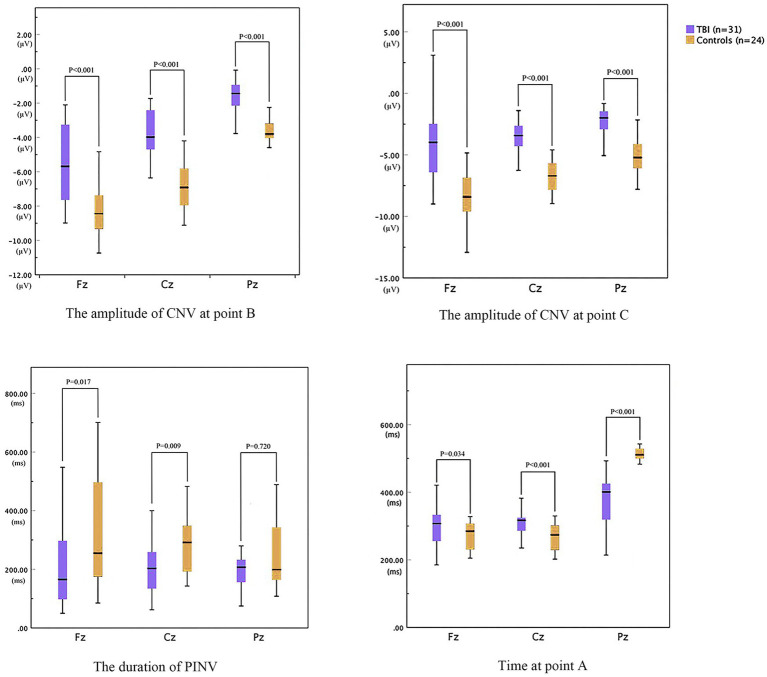
The parameters of CNV between patients with neurocognitive disorders after TBI and controls.

As shown in Figure 3, the time of point A in patients was later than that in healthy controls at electrodes Fz and Cz. In contrast, the outcome showed the opposite at electrode Pz. Compared to the control group, the TBI patient group exhibited significantly lower amplitudes at points B and C (*p* < 0.001), as shown in Table 2 and all three grand average EPR response figures. The topographic distribution also showed that the CNV amplitudes in the patient group were much lower than that in the control group, while the high-amplitude range was much smaller (Figure 4). Furthermore, the duration of PINV at Fz and Cz was significantly shorter in the TBI patient group than in the control group (*p* = 0.017 and *p* = 0.009, respectively). However, there was no statistically significant difference in the duration of PINV at Pz between the two groups (*p* = 0.720). The time at point A of Fz and Cz was significantly earlier in the control group than in the patient group (*p* = 0.034 and *p* < 0.001, respectively). In contrast, the time at point A of Pz was significantly earlier in the patient group (*p* < 0.001) (Figure 5).

**Table 2:** The results of CNV between patients with neurocognitive disorders after TBI and controls.

Parameter	TBI (*n* = 31)	Controls (*n* = 24)	Value of *p*
Amplitude B (μV)			
Fz	−(5.55 ± 2.25)	−(8.21 ± 1.69)	<0.001^a^
Cz	−(3.82 ± 1.46)	−(6.86 ± 1.33)	<0.001^a^
Pz	−(1.63 ± 1.10)	−(3.68 ± 0.79)	<0.001^a^
Amplitude C (μV)			
Fz	−(4.45 ± 2.77)	−(8.15 ± 2.01)	<0.001^a^
Cz	−(3.61 ± 1.42)	−(6.80 ± 1.23)	<0.001^a^
Pz	−(2.21 ± 1.02)	−(5.06 ± 1.53)	<0.001^a^
Duration of PINV (ms)			
Fz	219.11 ± 160.43	337.39 ± 203.15	0.017^a^
Cz	210.65 ± 88.69	282.75 ± 100.52	0.009^a^
Pz	227.41 ± 116.34	246.33 ± 114.37	0.720
Time at point A (ms)			
Fz	302.82 ± 62.27	270.04 ± 41.16	0.034^a^
Cz	308.32 ± 36.56	265.33 ± 39.09	<0.001^a^
Pz	370.22 ± 86.22	493.88 ± 98.20	<0.001^a^

TBI, traumatic brain injury. Data were presented as mean ± SD. Data were calculated by Student’s *t*-test. ^a^Statistically significant differences.

#### Correlations between CNV and scale scores

As shown in Table 3, in the TBI patient group, the time at point A of Fz was positively correlated with ADL, FAQ, SDSS, and personality change assessment scale scores (*p* < 0.05). The time at point A of Pz was negatively correlated with FAQ, SDSS, and personality change assessment scale scores (*p* < 0.05). However, no other statistically significant correlations were found between the time at point A and scale scores. Additionally, both amplitudes B and C were positively correlated with four scale scores (*p* < 0.05). The duration of PINV at Fz was negatively correlated with FAQ scale scores (*p* = 0.024). This negative correlation was also found between the duration of PINV at Cz and ADL, SDSS, and personality change assessment scale scores (*p* = 0.042, *p* = 0.047, and *p* = 0.004, respectively). There was no other correlation between PINV duration and scale assessment scores (*p* > 0.05).

**Table 3:** Correlations between CNV and scale scores in patients with neurocognitive disorders after TBI.

Parameter	ADL scale		FAQ scale		SDSS scale		Personality change scale	
	Value of *p*	*r*-value	value of p	r-value	value of p	r-value	value of p	r-value
Amplitude B (μV)								
Fz	0.005^a^	0.385	0.001^a^	0.45	0.001^a^	0.466	0.023^a^	0.328
Cz	<0.001^a^	0.489	<0.001^a^	0.557	<0.001^a^	0.529	<0.001^a^	0.527
Pz	<0.001^a^	0.48	<0.001^a^	0.491	0.001^a^	0.469	<0.001^a^	0.544
Amplitude C (μV)								
Fz	0.002^a^	0.418	0.001^a^	0.437	0.002^a^	0.433	<0.001^a^	0.499
Cz	0.001^a^	0.458	0.001^a^	0.612	<0.001^a^	0.489	<0.001^a^	0.508
Pz	0.004^a^	0.395	<0.001^a^	0.491	0.001^a^	0.442	0.001^a^	0.475
Duration of PINV (ms)								
Fz	0.095	−0.236	0.024^a^	−0.315	0.065	−0.26	0.050	−0.284
Cz	0.042^a^	−0.276	0.052	−0.263	0.047^a^	−0.269	0.004^a^	−0.394
Pz	0.439	−0.111	0.705	−0.054	0.314	−0.144	0.362	−0.135
Time at point A (ms)								
Fz	0.001^a^	0.461	<0.001^a^	0.477	<0.001^a^	0.496	<0.001^a^	0.493
Cz	0.402	0.115	0.079	0.239	0.11	0.218	0.077	0.248
Pz	0.066	−0.26	0.002^a^	−0.418	0.012^a^	−0.348	0.010^a^	−0.367

ADL, activity of daily living; FAQ, functional activities questionnaire; SDSS, social disability screening schedule; TBI, traumatic brain injury. Data were calculated by Pearson’s correlation analysis. ^a^Statistically significant differences.

## Discussion

Numerous factors are linked to the severity of mental disorders, yet assessing their correlations can be challenging. The subcomponents of ERPs are regarded as ideal markers to evaluate brain function, indicating the severity of cognitive impairment. While most research has focused on P300 and MMN abnormalities after TBI, previous studies reported CNV abnormalities in TBI patients compared to healthy controls (24). Current study focused on using CNV to evaluate neurocognitive disorder and its severity after TBI. Our results indicate that TBI patients have significantly higher ADL, FAQ, SDSS, and personality change scale scores than the control group, suggesting that TBI might accompany neurocognitive disorders and patients after a TBI might suffer from social dysfunctions and personality disorders.

A lower amplitude of the pre-response CNV component is related to the reaction time (RT) in patients with TBI, which is correlated with electrophysiological measures of attention and information processing (25). Additionally, late amplitude is smaller in mTBI patients than in healthy controls (26). Therefore, it was presumed that decreased CNV amplitude could be an indicator reflecting a psychotic state or attention problems resulting from suboptimal cortical excitability (27). The negative level of CNV was related to the interval time between two stimuli and repetition enhancement, indicating the influence of decisional and memory mechanisms on CNV and a lager amplitude is associated with better behavior (28, 29). In our study, the TBI patient group had significantly lower amplitudes at points B and C compared to the control group, which is consistent with previous research mentioned before.

PINV reflects the period during which the negative wave returns to baseline after S2 and the duration of PINV may differ depending on different impairment symptoms. Our findings showed that the duration of PINV was significantly shorter at Fz and Cz in the TBI patient group compared to the control group, indicating neurocognitive impairment symptoms after TBI. The decrease in the duration of PINV and the amplitude might suggest a lower level of arousal and attention. However, there was no statistically significant difference in the duration of PINV at Pz between the two groups. Furthermore, our result showed that the time at point A of Fz and Cz was significantly shorter in the control group than in the patient group, while the time at point A of Pz was significantly shorter in the patient group. The delay in latency at point A was also a significant sign of inattention.

TBI is associated with the impairment of daily life ability and social function (30). Recent studies have also linked abnormal CNV late amplitude with motor manifestations in Parkinson’s disease, conduct disorder, and antisocial personality traits (31, 32). Although the accurate reason for personality change after a TBI remains unknown, several factors, such as dysfunction of the frontal lobe, were suggested to be associated with personality change (33). In this study, we aimed to assess brain impairment after TBI more comprehensively by exploring the correlation between CNV parameters and daily life ability, as well as social function, through assessment scales. ADL, FAQ, SDSS, and personality change scale scores were significantly higher in the TBI patient group than in the control group, indicating the impairment of daily life and social function after a TBI. According to our study, lower amplitudes of CNV at points B and C were associated with more severe neurocognitive disorder and greater impairment of daily life ability and social function. As mentioned before, this correlation might result from impairment of information processing, arousal, and attention. Personality change is also regarded as a main symptom of neurocognitive disorder after a TBI, which is correlated with the impairment of daily life ability and social function. Lower amplitudes of CNV at points B and C, a shorter duration of PINV at Cz, and a delay in latency at point A meant a more severe level of personality change according to our result. Therefore, our study assumed that CNV parameters could also reflect a personality disorder to some extent, indicating that CNV might be used as an indicator to reveal a personality change and assess its severity after a TBI.

### Limitations

In this study, several limitations should be acknowledged. First, the sample size was relatively small, which might have led to statistical bias resulting from individual differences. Second, TBI patients’ symptoms were comparatively mild due to consideration of the subjects’ cooperation level. Third, we did not analyze the specific type of brain injury, as the primary focus of this study was to explore the association between CNV parameters and the severity of neurocognitive disorder following a TBI. Additionally, only three individual electrodes were used in this study. If a more precise correlation between CNV and the severity of mental disorders following a TBI is sought, future studies should select more electrodes and contain a larger sample size. Considering that mTBI is more difficult to detect and the cognitive impairment of mTBI may differ compared to severe or moderate TBI, future studies should categorize TBI according to the different severities of TBI as well as the type of brain injury.

## Conclusion

Patients with neurocognitive disorder following a TBI often experience impairments in daily life abilities, social function, and personality. Our study found that lower amplitudes of CNV at points B and C were associated with more severe neurocognitive disorder and greater impairments in daily life abilities and social function. Additionally, the duration of PINV and the latency of point A were correlated with the neurocognitive disorder to some extent. Personality changes and their severity could be revealed through CNV parameters. In conclusion, CNV has the potential to be used as an objective, electrophysiology-based parameter for evaluating the severity of neurocognitive disorder and personality changes after a TBI.

## Data availability statement

The raw data supporting the conclusions of this article will be made available by the authors, without undue reservation.

## Ethics statement

The studies involving humans were approved by ethics committee of the Academy of Forensic Science. The studies were conducted in accordance with the local legislation and institutional requirements. The participants provided their written informed consent to participate in this study.

## Author contributions

XL: Conceptualization, Formal Analysis, Writing – original draft. SW: Investigation, Writing – original draft. SZ: Investigation, Formal Analysis. WL: Investigation, Formal Analysis. QZ: Investigation, Supervision. WC: Supervision, Writing – review & editing. HL: Conceptualization, Supervision, Writing – original draft, Writing – review & editing.

## References

[1] Bruns J Jr Hauser WA. The epidemiology of traumatic brain injury: a review. Epilepsia. (2003) 44:2–10. doi: 10.1046/j.1528-1157.44.s10.3.x 14511388

[2] Livingston G Huntley J Sommerlad A Ames D Ballard C Banerjee S. Dementia prevention, intervention, and care: report of the lancet commission. Lancet. (2020) 396:413–46. doi: 10.1016/S0140-6736(20)30367-6, PMID: 32738937 PMC7392084

[3] Fann JR Burington B Leonetti A Jaffe K Katon WJ Thompson RS. Psychiatric illness following traumatic brain injury in an adult Healthmaintenance organization population. Arch Gen Psychiatry. (2004) 61:53–61. doi: 10.1001/archpsyc.61.1.53, PMID: 14706944

[4] Dewan MC Rattani A Gupta S Baticulon RE Hung Y-C Punchak M. Estimating the global incidence of traumatic brain injury. J Neurosurg. (2018) 130:1080–97. doi: 10.3171/2017.10.JNS17352 29701556

[5] Gudigar A Raghavendra U Hegde A Menon GR Molinari F Ciaccio EJ. Automated detection and screening of traumatic brain injury (Tbi) using computed tomography images: a comprehensive review and future perspectives. Int J Environ Res Public Health. (2021) 18:6499. doi: 10.3390/ijerph18126499, PMID: 34208596 PMC8296416

[6] Pavlovic D Pekic S Stojanovic M Popovic V. Traumatic brain injury: neuropathological, neurocognitive and neurobehavioral sequelae. Pituitary. (2019) 22:270–82. doi: 10.1007/s11102-019-00957-9, PMID: 30929221

[7] Toth A. Magnetic resonance imaging application in the area of mild and acute traumatic brain injury: Implications for diagnostic markers? In: Firas HK, editors. Brain Neurotrauma: Molecular, Neuropsychological, and Rehabilitation Aspects. Boca Raton, FL: CRC Press/Taylor & Francis (2015) 26269902

[8] Walter WG Cooper R Aldridge V McCallum W Winter A. Contingent negative variation: an electric sign of Sensori-motor association and expectancy in the human brain. Nature. (1964) 203:380–4. doi: 10.1038/203380a0 14197376

[9] Rohrbaugh JW. The orienting reflex: performance and central nervous system manifestations In: Parasuraman R Davies R, editors. Varieties of attention. Orlando, FL: Academic Press (1984). 323–73.

[10] Zhang X Liu X Wang Y Liu C Zhang N Lu J. Exploration of cortical inhibition and habituation in insomnia: based on Cnv and Eeg. Methods. (2022) 204:73–83. doi: 10.1016/j.ymeth.2022.01.012, PMID: 35121141

[11] Wen D Cheng Z Li J Zheng X Yao W Dong X. Classification of Erp signal from amnestic mild cognitive impairment with type 2 diabetes mellitus using single-scale multi-input convolution neural network, 363:109353. J Neurosci Methods. (2021) 363:109353. doi: 10.1016/j.jneumeth.2021.109353, PMID: 34492241

[12] Showkath N Sinha M Ghate JR Agrawal S Mandal S Sinha R. Eeg-Erp correlates of cognitive dysfunction in polycystic ovarian syndrome. Ann Neurosci. (2022) 29:09727531221115318:225–32. doi: 10.1177/09727531221115318, PMID: 37064285 PMC10101155

[13] Li H Li N Xing Y Zhang S Liu C Cai W. P300 as a potential Indicator in the evaluation of neurocognitive disorders after traumatic brain injury. Front Neurol. (2021) 12:690792. doi: 10.3389/fneur.2021.690792, PMID: 34566838 PMC8458648

[14] Ganapathi AS Glatt RM Bookheimer TH Popa ES Ingemanson ML Richards CJ. Differentiation of subjective cognitive decline, mild cognitive impairment, and dementia using qEEG/ERP-based cognitive testing and volumetric MRI in an outpatient specialty memory clinic. J Alzheimers Dis. (2022) (Preprint):1-9) 90:1761–9. doi: 10.3233/JAD-220616, PMID: 36373320 PMC9789480

[15] Donaldson KR Jonas K Foti D Larsen EM Mohanty A Kotov R. Mismatch negativity and clinical trajectories in psychotic disorders: five-year stability and predictive utility. Psychol Med. (2022) 53:5818–28. doi: 10.1017/S0033291722003075, PMID: 36226640 PMC10782876

[16] Hansenne M Ansseau M. Contingent negative variation and personality in depression. Neuropsychobiology. (2001) 44:7–12. doi: 10.1159/000054907 11408786

[17] Roth WT Duncan CC Pfefferbaum A Timsit-Berthier M. Applications of cognitive ERPs in psychiatric patients. Electroencephalogr Clin Neurophysiol Suppl. (1986) 38:419–38. PMID: 3466780

[18] Giedke H Bolz J Heimann H. Evoked potentials, expectancy wave, and skin resistance in depressed patients and healthy controls. Pharmakopsychiatr Neuropsychopharmakol. (1980) 13:91–101. doi: 10.1055/s-2007-1019618 7394000

[19] van den Bosch RJ Rozendaal N Mol JM. Slow potential correlates of frontal function, psychosis, and negative symptoms. Psychiatry Res. (1988) 23:201–8. doi: 10.1016/0165-1781(88)90010-8, PMID: 3363028

[20] Duncan CC Summers AC Perla EJ Coburn KL Mirsky AF. Evaluation of traumatic brain injury: brain potentials in diagnosis, function, and prognosis. Int J Psychophysiol. (2011) 82:24–40. doi: 10.1016/j.ijpsycho.2011.02.013 21356253

[21] Wilson L Horton L Polinder S Newcombe VF Nv S Maas AI. Tailoring multi-dimensional outcomes to level of functional recovery after traumatic brain injury. J Neurotrauma. (2022) 39:1363–81. doi: 10.1089/neu.2022.0013, PMID: 35607855

[22] Li X Gao B Wu D Liang W Ding S. Quantitative evaluation of personality changes induced by Craniocerebral injury. Chin J Clin Rehabil. (2006) 10:10–2. doi: 10.12307/j.issn.2095-4344.2006.06.004

[23] Fan H-Y Zhang Q Tang T Cai W. Personality change due to brain trauma caused by traffic accidents and its assessment of psychiatric impairment. Fa Yi Xue Za Zhi. (2016) 32:100–4. doi: 10.3969/j.issn.1004-5619.2016.02.006, PMID: 27501680

[24] Rizzo P Amabile G Caporali M Spadaro M Zanasi M Morocutti C. A CNV study in a Group of Patients with traumatic head injuries. Electroencephalogr Clin Neurophysiol. (1978) 45:281–5. doi: 10.1016/0013-4694(78)90012-3 78838

[25] Bender S Weisbrod M Bornfleth H Resch F Oelkers-Ax R. How do children prepare to react? Imaging maturation of motor preparation and stimulus anticipation by late contingent negative variation. NeuroImage. (2005) 27:737–52. doi: 10.1016/j.neuroimage.2005.05.020 16027009

[26] Müller A Candrian G Dall’Acqua P Kompatsiari K Baschera G-M Mica L. Altered cognitive processes in the acute phase of MTBI: an analysis of independent components of event-related potentials. Neuroreport. (2015) 26:952–7. doi: 10.1097/WNR.0000000000000447, PMID: 26317478

[27] Kimble M Ruddy K Deldin P Kaufman M. A CNV-distraction paradigm in combat veterans with posttraumatic stress disorder. J Neuropsychiatry Clin Neurosci. (2004) 16:102–8. doi: 10.1176/jnp.16.1.102, PMID: 14990765

[28] Wiener M Thompson JC. Repetition enhancement and memory effects for duration. NeuroImage. (2015) 113:268–78. doi: 10.1016/j.neuroimage.2015.03.054, PMID: 25818689

[29] Gontier E Hasuo E Mitsudo T Grondin S. EEG investigations of duration discrimination: the intermodal effect is induced by an attentional Bias. PLoS One. (2013) 8:e74073. doi: 10.1371/journal.pone.0074073 24009766 PMC3751868

[30] Whitnall L McMillan T Murray G Teasdale G. Disability in young people and adults after head injury: 5–7 year follow up of a prospective cohort study. J Neurol Neurosurg Psychiatry. (2006) 77:640–5. doi: 10.1136/jnnp.2005.078246, PMID: 16614025 PMC2117429

[31] Guan M Ma L Zhu Y Liao Y Zeng L Wu S. Impaired sustained attention in groups at high risk for antisocial personality disorder: a contingent negative variation and standardized low-resolution tomographic analysis study. Front Hum Neurosci. (2022) 16:925322. doi: 10.3389/fnhum.2022.925322, PMID: 36504621 PMC9726724

[32] Tzvetanov P Lisichkov I Rousseff RT Hegde V Kostadinov S. Abnormality of contingent negative variation correlates with Parkinson’s disease severity. Innovat Clin Neurosci. (2022) 19:71–6. Available at: https://www.ncbi.nlm.nih.gov/pmc/articles/PMC9507138/PMC9507138 36204175

[33] Chow TW. Personality in frontal lobe disorders. Curr Psychiatry Rep. (2000) 2:446–51. doi: 10.1007/s11920-000-0031-5, PMID: 11122995 PMC5786154

